# Data Sources and Analytic Approaches Used to Evaluate the Impact of Patient and Public Involvement in Child Health Research: Simple Random Survey of Individual Studies in Published Reviews

**DOI:** 10.1111/hex.70577

**Published:** 2026-02-20

**Authors:** Isabella Miklaucic, Cornelia M. Borkhoff, Sadia Akbar, Francine Buchanan, Nayantara Hattangadi, Antonia Giannarakos, Susan Law, Colin Macarthur

**Affiliations:** ^1^ Child Health Evaluative Sciences, SickKids Research Institute Toronto Ontario Canada; ^2^ Division of Paediatric Medicine and Paediatric Outcomes Research Team (PORT) The Hospital for Sick Children Toronto Ontario Canada; ^3^ Institute of Health Policy, Management & Evaluation, Dalla Lana School of Public Health, University of Toronto Toronto Ontario Canada; ^4^ Dalla Lana School of Public Health, University of Toronto Toronto Ontario Canada; ^5^ Library & Knowledge Services, Trillium Health Partners Mississauga Ontario Canada; ^6^ Department of Paediatrics Temerty Faculty of Medicine, University of Toronto Toronto Ontario Canada

**Keywords:** child health research, evaluation, patient and public involvement, patient‐oriented research, simple random survey

## Abstract

**Introduction:**

Several systematic and scoping reviews have focused on the impact of patient and public involvement (PPI) in child health research. The aim of this research was to examine the data sources and analytic approaches used by researchers to evaluate the impact of PPI in child health research.

**Methods:**

A comprehensive literature search identified published reviews focused on PPI impact in child health research. Titles, abstracts, and full texts were screened to determine eligibility. Individual studies from eligible reviews created the sampling frame, from which 100 individual studies were randomly selected. Information was extracted on primary study characteristics, type of PPI, data sources and analytic approaches used to evaluate PPI impact, and reported PPI impact. Frequency distributions were used to summarise the findings.

**Results:**

The initial search yielded 5868 citations. After screening, 15 reviews (comprising 406 individual studies, of which 303 were unique) met inclusion criteria. Among the 100 randomly selected individual studies, PPI was reported across all phases of the research process including priority setting (30/100), input on study materials (50/100), and dissemination of study findings (52/100). The method of PPI was most commonly focus groups (29/100) or advisory committees (27/100). Of the 100 studies selected, 67/100 reported impact on the research process, 69/100 reported impact on patients and families, and 32/100 studies reported impact on researchers. Regarding the data sources used to evaluate PPI impact, 69/100 studies reviewed primary study field notes along with researcher observations and reflections, while 31/100 studies conducted independent, specific, focus groups and/or interviews and/or surveys to gather data. Regarding the analytic approaches used to evaluate PPI impact, formal qualitative and/or quantitative analyses of the data occurred in only 25/100 studies. Only 2/100 studies used a formal PPI reporting guideline.

**Conclusion:**

Only 31/100 studies collected specific, independent data on the impact of PPI in child health research, and only 25/100 studies applied formal analyses. Robust evaluation of PPI impact in child health research is essential for a strong PPI evidence base.

**Patient or Public Contribution:**

A patient partner (co‐author, FB) was a member of the research team from study inception, and contributed to the development of the study protocol, including refining the research question, input on study design, selection of relevant outcomes, interpretation of findings, writing the manuscript, and developing dissemination plans.

## 1 Introduction

Patient and public involvement (PPI) in health and social research is an international movement, with national PPI initiatives launched in the United Kingdom (INVOLVE), Canada (Strategy for Patient‐Oriented Research), and the United States (Patient‐Centered Outcomes Research Institute, PCORI) over the last several decades [1, 2, 3]. PPI in health research has been defined as “research being carried out ‘with’ or ‘by’ members of the public rather than ‘to’, ‘about’, or ‘for’ them” [4]. Implicit goals of PPI are to prioritise and conduct research that is relevant, meaningful, and acceptable to the public, increase dissemination and uptake of research findings, and ultimately influence practice, policy, and health systems [5]. For example, the aim of Canada's Strategy for Patient‐Oriented Research (SPOR) is for “patients, researchers, health care providers and decision makers to actively collaborate to build a sustainable, accessible, and equitable health care system to bring about positive changes in the health of people living in Canada” [6].

There remains, however, skepticism among some researchers about the value of PPI in health research [7, 8, 9]. For example, Esmail et al. [9] noted that while numerous benefits of PPI in research are postulated, relatively few papers provide empirical data on benefits, with the evidence base often consisting of anecdotal evidence or researcher perception of impact. Identified barriers to the evaluation of PPI in health research include varied terminology for PPI, inconsistent reporting of PPI, no standardised evaluation framework, few valid measurement tools, a predominant focus on the ‘process’ of PPI rather than on ‘outcomes,’ and the lack of quantitative data on impact of PPI [8, 9, 10, 11, 12].

Several systematic and scoping reviews have reported the perceived benefits of PPI in child health research on the research process, researchers, and patient partners [13, 14, 15]. There is limited research, however, on the data sources and analytic approaches used to generate the evidence base regarding the impact of PPI in child health research. Therefore, the objective of this research was to examine individual studies included in published reviews focused on the impact of PPI in child health research to determine the range of data sources and analytic approaches used to formally measure the impact of PPI in child health research. *A priori*, “impact” was broadly defined as any “effect” (positive or negative) ‐ as reported by the authors of published manuscripts ‐ on the research process, researchers, or patient partners as a result of the involvement of patients, families, caregivers, or the public in research.

## 2 Materials and Methods

### 2.1 Search Strategy

A comprehensive search strategy to identify all published reviews focused on the impact of PPI in child health research was developed with the assistance of a health research librarian (AG). The following databases were searched using a search strategy based on the 2015 Peer‐Review of Electronic Search Strategies (PRESS) Guidelines [16]: Ovid MEDLINE, Ovid Embase, Cochrane Database of Systematic Reviews Ovid, PsycINFO Ovid, Elsevier Scopus, EBSCO CINAHL, and PubMed Non‐Medline records. Medical subject headings, Emtree terms, CINAHL subject headings, and free text words related to patient and family engagement and pediatric or child health research were used to retrieve relevant records. The search was restricted to English language articles published from January 2009 to July 2024. The Year 2009 was chosen as the starting point for the literature review, given that several national PPI in health research initiatives were launched around that time, such as PCORI in the US in 2010 and SPOR in Canada in 2011 [17, 18]. The Ovid MEDLINE search strategy, which formed the basis for the search strategies for the other electronic databases, is shown in Supporting Information S1: Table S1.

### 2.2 Study Design, Sampling Frame, and Data Abstraction

A simple random survey design was used. The literature search identified systematic, scoping, narrative, critical, mixed‐methods, rapid, qualitative, integrated, realist, and state‐of‐the‐art reviews, and meta‐analyses on PPI in child health research. Published reviews that did not include at least one article evaluating the impact of PPI on child health research were excluded. As described earlier, for the purpose of this study, PPI was defined as the authentic involvement of patients, family, or community members at any stage throughout the research process, e.g., to inform research priorities, study design, conduct, or dissemination [4, 5]. The research population of interest was children under 18 years of age. Health research was broadly defined to encompass all four pillars of the Canadian Institutes of Health Research, namely, biomedical, clinical, health services, and population health [19].

Titles, abstracts, and full texts of potentially eligible reviews were screened by two reviewers (IM, SA). Following the identification of all eligible reviews, the individual studies included in these reviews were combined to create the sampling frame. Next, after removal of duplicates, simple random sampling was used to select the full text of 100 individual studies that evaluated the impact of PPI in child health research. A random sample of 100 studies was chosen as we felt that a sample of 1/3 of the sampling frame (*n* = 303) would be sufficient to generalise results. Further, based on an arbitrary *a priori* assumption that 25% of studies would use formal analytic approaches to evaluate PPI impact, a sample of 100 studies would provide a 95% confidence interval of +/− 8% around the point estimate.

A standardised data abstraction form (Supporting Information S2: Table S2)—informed by the Guidance for Reporting Involvement of Patients and the Public (GRIPP2) reporting checklist—was developed *a priori* [20]. Data were collected on (a) primary study PPI characteristics (group, timing, method of engagement); (b) data sources and analytic approaches used to determine the impact of PPI; and (c) impact of PPI in child health research on three domains (research conduct, researchers, and PPI partners) as reported in the published manuscripts. An *a priori* list of potential PPI impacts was derived from the relevant literature [13]. Impacts were categorised based on this list, as well as inductively added to the list as novel impacts emerged from the data. Frequency distributions were used to summarise the findings.

## 3 Results

**Figure 1:**
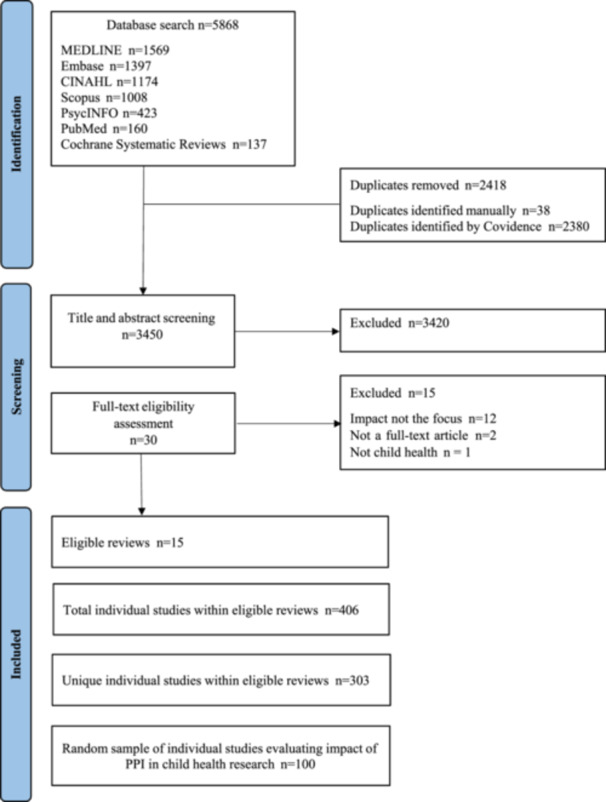
PRISMA flow diagram.

Figure 1 describes the search results and screening process based on PRISMA reporting guidelines [21]. The initial literature search yielded a total of 5868 citations. After removing duplicates (*n* = 2418), the titles and abstracts of 3450 citations were screened for eligibility by IM (with full agreement on eligibility on a 10% sample screened by SA). In total, 30 full‐text publications were reviewed (IM and SA), with 15 published reviews meeting inclusion criteria. These 15 reviews (Supporting Information S3: Table S3) comprised a total of 406 individual studies. After removal of duplicates, 303 unique individual studies remained. Of these 303 peer‐reviewed, published articles that examined the impact of PPI in child health research, 100 papers were randomly selected using a random number generator (Supporting Information S4: Table S4).

Table 1 describes the PPI characteristics of these 100 studies. Youth were the most frequently engaged partner (85/100), with stakeholders engaged in 42/100 studies. Stakeholders included parents, guardians, siblings, and community members (including citizens, professional advocacy group members, and educators). Of the 42 studies engaging stakeholders, the most common stakeholders were parents (29/42) and community members (15/42). Focus groups (29/100) and advisory committees (27/100) were the most common method of PPI. Studies reported PPI across all phases of the research process—key areas of PPI included priority setting (30/100), input on study materials (50/100), and dissemination of study findings (52/100).

**Table 1:** PPI characteristics of primary studies (*N* = 100).

Characteristic	Number
**Individuals engaged**	
Children (0–11 years)	2
Youth (12–17 years)	50
Children + youth (0–17 years)	6
Stakeholders^a^ only	12
Stakeholders + youth	23
Stakeholders + children + youth	6
Stakeholders + children	1
**Number engaged**	
1–5	9
6–20	49
21–99	29
100+	4
Not reported	9
**Method of engagement** b	
Patient‐led research	21
Member of research team	19
Advisory committee	27
Focus group	29
Interview	15
Surveys/written feedback	3
**Area of engagement** b	
Priority setting	30
Development of research proposal	10
Funding application	6
Development of the research question	37
Selection of outcomes	18
Intervention development and refinement	23
Input on study design	60
Input on study conduct and feasibility	27
Input on study materials	50
Input on recruitment of participants	22
Intervention delivery	15
Data collection	51
Data analysis and interpretation	63
Drafting manuscript	13
Dissemination and implementation of findings	52

^a^ Stakeholders included parents, guardians, siblings, citizens, professional advocacy group members, educators.

^b^ More than one option per study possible.

Table 2 describes the data sources and the analytic approaches used to evaluate the impact of PPI in child health research, across the 100 individual studies. Data sources included reviewing field notes, researcher observations and reflections, independent focus groups, in‐person interviews, and specific surveys. Field notes were notes that researchers wrote during the research process and were often described in such terms, whereas observations and reflections were less systematically recorded, with little information provided about when or how they were documented. For 69/100 studies, a review of the primary study field notes along with researcher observations and reflections were the sole data sources used to determine the impact of PPI. Formal, independent data collection to evaluate PPI impact occurred in 31/100 studies: 18/30 studies held specific focus groups and/or interviews, 8/100 studies held specific focus groups/interviews and conducted specific surveys, and 5/100 studies conducted stand‐alone, specific surveys to gather data on the impact of PPI in child health research.

**Table 2:** Data sources and analytic approaches used to evaluate PPI impact in primary studies.

	# of Studies (*N* = 100)
**Data sources used to gather data on PPI Impact**	
Primary study data (*n* = 69)	
Field notes/researcher observations and reflections	69
Independent data collection (*n* = 31)	
Focus groups/in‐person interviews	18
Focus groups/in‐person interviews/surveys	8
Surveys	5
**Analytic approaches used to evaluate PPI Impact**	
Descriptive categories based on researcher perceptions	55
Descriptive categories based on patient partner perceptions	3
Descriptive categories based on researcher and patient partner perceptions	17
Qualitative (thematic) analysis	22
Mixed‐methods (quantitative and qualitative analysis)	3

Regarding the analytic approaches used for the data collected, no formal analysis, i.e., simple descriptive categorisation of PPI impact across the three domains (on the research process, on researchers, and on patient partners)—as reported by authors—was the most common analytic approach (75/100). Descriptive categories were often presented in an unstructured format such as narrative text in the Results and Discussion sections of the published papers. Formal qualitative analysis (thematic and content) of focus group transcripts, in‐person interviews, and survey data was used to measure PPI impact in 22/100 studies. A combination of quantitative and qualitative analysis (mixed‐methods) was used to evaluate PPI impact in 3/100 studies. In summary, formal qualitative and/or quantitative analysis occurred in 25/100 studies (25%; 95% confidence interval: 17%–35%). Of note, only two studies measuring PPI impact referenced the GRIPP2 reporting framework [20]. No other reporting guidelines or evaluation tools were referenced.

Table 3 describes the reported impacts of PPI in child health research. Most studies (67/100) reported PPI impacts on the research process, including improving the usefulness and relevance of research findings (32/100), co‐production of study materials to make them appropriate for the study population (25/100), and helping to identify and prioritise important and relevant topics for research (23/100). Impacts on researchers were reported in 32/100 studies and included improved relationships between researchers and patients/families (16/100), broadened perspectives (13/100), and enhanced knowledge and skills (10/100). Reported impacts on patients and families (69/100) included increased knowledge and skills (47/100), empowerment (44/100), and enhanced career and academic opportunities (23/100).

**Table 3:** Reported impact of PPI in child health research.

	# of Studies
**Impact on research process** a **(*N*** = **67/100)**	
Improving the usefulness/relevance of findings	32
Co‐producing study materials appropriate for patient population	25
Helping to identify/prioritise important/relevant topics for research	23
Increasing participant recruitment/retention	15
Enhanced analysis	14
Improving design of research	12
Refining the research question	8
Improving research efficiency	6
Enhanced dissemination and implementation of research	6
Improving research quality	5
Helping to secure funding	3
**Impact on the research team** a **(*N*** = **32/100)**	
Improved relationships between researchers and patients/families	16
Gaining a broader perspective	13
Enhanced knowledge/skills	10
Cultural competency/change in attitudes towards patient population	5
Improved provision of care	4
Increased need for resources	4
Networking/community building	1
Improved satisfaction	1
**Impact on patients and families** a **(*N*** = **69/100)**	
Increase in knowledge and skills	47
Empowerment/recognition of patient partner influence	44
Networking/career and academic outcomes	23
Community building	20
Improved relationships between patients/families and researchers	10
Improved care experience and understanding of clinical condition	10
Increased resilience/independence	10
Gaining a broader perspective	9
Increased awareness of need for resources	2
Improved relationship/understanding between parents and children	2

^a^ More than one option per study possible.

## 4 Discussion

This simple random survey of published articles included in reviews on the impact of PPI in child health research found numerous reported PPI impacts on the research process, researchers, and patient partners. The range of data sources used in the selected studies to gather information on PPI impact included reviews of field notes, researcher observations and reflections, focus groups, interviews, and surveys. Only 31/100 studies collected formal, independent data—through specific focus groups, interviews, and surveys—to evaluate PPI impact. Formal analysis (qualitative or quantitative) of the gathered data to determine impact occurred in only 25/100 studies, and only 2/100 articles referenced the GRIPP2 reporting guidelines. The analytic approach for the remaining studies (75/100) was to simply describe PPI impact in terms of the perceptions of researchers and patient partners. Unstructured, narrative reporting of researcher and patient partner perceptions of impact, while useful, are not particularly robust outcome measures, given the potential for conflicts of interest and the risk of ‘confirmation bias,’ i.e., reporting a positive effect of PPI, given *a priori* beliefs (by researchers and/or patient partners) in the value of PPI.

A long‐recognised challenge for PPI in health research has been the “quality and utility” of the evidence base [10, 20, 22]. In 2011, Stanizewska et al. [10] noted that the evidence base for PPI in research “remains partial and often lacks coherence,” with poor conceptualisation of engagement, limited theoretical work, few quantitative data, and poor reporting identified as challenges. Development of an evaluation instrument—based on a theoretical model or framework—that captured all dimensions of PPI impact, measured the extent of impact, and provided data on impact on different groups and at different stages of the research process was considered an important next step. The Guidance for Reporting Involvement of Patients and the Public (GRIPP2)—an evidence‐informed, international consensus measure that identifies the key items to report to enhance the quality, transparency, and consistency of PPI in the health research evidence base—emerged from this work [20]. Despite the availability of GRIPP2, reporting of PPI is inconsistent [23]. Failure to report PPI in published papers may be related to the lack of standardised reporting, the optional nature of reporting PPI in manuscripts, and word count limitations for authors [8, 23]. Of note, 22/100 selected articles were published prior to the publication of the original GRIPP guidelines in 2011 and 66 were published before the GRIPP2 in 2017, which may account for the infrequent use of GRIPP/GRIPP2 in our sample.

Several authors have examined the feasibility of measuring PPI impact. For example, Barber et al. [24] conducted a sequential mixed‐methods study (two‐round Delphi survey and interviews) involving researchers, citizens, research managers, and policy makers. Participants agreed that it would be feasible (“it can be done”) to evaluate the impact of PPI on identifying research topics, setting research priorities, dissemination of research findings, and impact on research team members and on patient partners. Dillon et al. [25] hosted a workshop involving 24 researchers and 5 patient partners to identify key research outcomes and associated measures to evaluate the impact of patient engagement. The workshop identified a series of critical outcomes of research engagement, such as whether the research was patient‐centred, meaningful, and rigorous, and a series of measures that could potentially map to these constructs.

Comprehensive evaluation frameworks for PPI in health research have been published. For example, Aubin et al. [8] used a Canadian Academy of Health Sciences report to develop a framework to measure the impact of patient‐oriented research. The framework measures the impact of patient‐oriented research in terms of value to patients and to researchers, improvements to the research process, impact on policies, impact on health outcomes, and contributions to social change in health research. While the framework is conceptually coherent, Aubin noted the difficulty in isolating the specific impact of the PPI component of a research study, given that many other factors, for example, inadequate resources for evaluation, local culture, policies, and personalities, might confound the assessment of PPI impact, particularly for longer‐term outcomes. As noted by Esmail et al. [9], “longer‐term outcomes typically have complex causes that are difficult to trace back to one research study, let alone the engagement of patients or other stakeholders in the research enterprise”.

Boivin et al. [26] conducted a systematic review of the literature (1980–2016) to identify and appraise tools used to evaluate the impact of patient and public engagement in research and health system decision making. Evaluation tools were appraised based on four criteria: scientific rigor, patient and public perspective, comprehensiveness, and usability. In total, 27 evaluation tools were identified, with only 14 tools specific to research. Scientific rigor was modest—only 11% of the evaluation tools were based on a literature review, and only 7% included testing for reliability. Just over half of the tools (56%) had patient or public involvement in the design and development of the measurement tool, however, PPI was mainly at the pilot stage. Most of the evaluation tools focused on PPI context, process and perceived, self‐reported impact. The authors recommended that future tools to evaluate the impact of patient engagement should collect data from both patient partners and researchers, and encouraged evaluation of PPI impact by independent, external evaluators. As an example, the Public and Patient Engagement Evaluation Tool (PPEET) comprises questionnaires for organisations, participants, and projects to evaluate engagement capacity, experiences, and outcomes. Independent external evaluation of PPI impact may mitigate the inherent biases that may arise when impact is based on the perceptions of the researchers and patient partners involved [27].

Preston et al. [28] adapted the GRIPP2 Short Form to evaluate engagement of children and youth in research. Research involving children differs from that in adults, given the communication needs and ethical considerations surrounding consent and vulnerability for children and youth [28]. Consequently, evaluating the impact of PPI in child health research, which ideally would involve the child patient partners themselves, may require distinct methodological approaches and specific evaluation tools, compared to the evaluation of PPI impact in adult research. To our knowledge, there are no validated child‐specific PPI impact evaluation tools. It may be useful for future work to examine the associations (if any) between methodological approaches and data sources used in PPI evaluation and the range, type, and size of impact reported.

Both researchers and patient partners have a vested interest in a strong evidence base for PPI as evidence of benefit promotes the use of PPI in health research and supports best practice [26]. Boivin et al. [26] also suggested that evaluation of PPI should be integrated into a broader evaluation of research quality, relevance, and effect, and that evaluation efforts ought to be grounded in key principles including clarity, reflexivity, methodological rigor, transparency, pragmatism, and reciprocity. Key questions that underpin the need for rigorous evaluation are whether PPI that is ‘evaluated’ and shown to be ‘ineffective’ is because of a failure of theory, a lack of reliable and valid measurement instruments, a failure to adequately report PPI, or a failure of practice of PPI, including inadequate resources or implementation [8, 26].

## 5 Conclusion

The prioritisation and conduct of research that is important, relevant, feasible, and acceptable to those whose tax dollars fund much of the research enterprise and to whom the results matter most underpins the ‘democratic’ ideal of PPI in health research [5]. Therefore, measuring the impact of PPI in health research and developing a robust evidence base are key components of the practice of PPI. In our study, however, only 31/100 studies collected independent data to evaluate PPI impact and only 25/100 studies applied formal (qualitative or quantitative) analyses to measure impact. Further, only 2/100 articles referenced the GRIPP2 reporting guidelines.

Potential barriers to measuring PPI impact include varied terminology, inconsistent reporting, no standardised evaluation framework, few measurement tools with evidence of reliability and validity, and a predominant focus on the ‘process’ of PPI rather than on outcomes [8]. Nevertheless, there are several potential ‘solutions’ that may improve the evidence base regarding the impact of PPI in health research such as the use of GRIPP2 reporting guidelines to ensure comprehensive reporting of PPI; identification of key themes related to ‘evaluation and impact’ for patient partners; formal, independent, external, data collection to evaluate impact; rigorous qualitative and/or quantitative analyses of evaluation data; standardised evaluation approaches to allow comparison across studies; researcher and patient partner consensus on ‘meaningful’ evaluation frameworks; and mapping theory, research methods, analytic approaches, and data collection to the evaluation framework chosen. Such strategies would help move the evidence base away from perceptions of impact to more reliable, valid, and reproducible forms of evidence to support PPI in child health research.

## Author Contributions

**Isabella Miklaucic:** conceptualisation, investigation, writing – original draft, methodology, writing – review and editing, formal analysis, data curation. **Cornelia M Borkhoff:** conceptualisation, methodology, supervision, writing – review and editing. **Sadia Akbar:** methodology, data curation, writing – review and editing. **Francine Buchanan:** conceptualisation, methodology, writing – review and editing. **Nayantara Hattangadi:** methodology, data curation, writing – review and editing. **Antonia Giannarakos:** methodology, data curation, writing – review and editing. **Susan Law:** conceptualisation, methodology, writing – review and editing. **Colin Macarthur:** conceptualisation, methodology, supervision, writing – original draft, writing – review and editing.

## Funding

The authors received no specific funding for this work.

## Conflicts of Interest

The authors declare no conflicts of interest.

## Supporting information


**Supplementary Table 1:** Ovid MEDLINE Search Strategy.

**Supplementary Table 2:** Data Abstraction Form.

**Supplementary Table 3:** Eligible Reviews.

**Supplementary Table 4:** Citations for 100 primary studies randomly selected from 15 eligible reviews.

## Data Availability

The data that support the findings of this study are available from the corresponding author upon reasonable request.
