# Supplementary material for: Data Sources and Analytic Approaches Used to Evaluate the Impact of Patient and Public Involvement in Child Health Research: Simple Random Survey of Individual Studies in Published Reviews
Source: Health Expect. 2026 Feb 20;29(1):e70577. doi: 10.1111/hex.70577 (PMC12928104; doi:10.1111/hex.70577)
Supplement: Supplementary file 1 — Supplementary Table 1: Ovid MEDLINE Search Strategy. [file HEX-29-e70577-s002.docx]

**Supplementary Table 1.** Ovid MEDLINE Search Strategy

**Ovid MEDLINE(R) ALL <January 01, 2009 to July 08, 2024>**

<https://ovidsp.ovid.com/ovidweb.cgi?T=JS&NEWS=N&PAGE=main&SHAREDSEARCHID=67RWBEnIdPxHRjFxGMqJJh4QgJ9Aqb1xVWaIOSAa3cJLQTKvkAYbP1ogu4RJm0q57>

1 patient participation/ or community participation/ or Community-Based Participatory Research/ 53749

2 ((patient* or ex-patient* or famil* or parent* or mother* or father or caregiver* or care-giver* or guardian* or relative* or public or consumer* or user* or citizen* or child* or sibling* or communit*) adj2 (activation or empowerment or participa* or involv* or engag* or consult* or collaborat* or research or co-research* or coresearch* or co-investig* or coinvestig* or co-design* or codesign* or interven* or advisor* or panel* or partner* or contribut* or oriented or guide* or input* or workshop* or "focus group*")).tw,kf. 525840

3 (Parent-led or family-led or patient-led or patient-driven or parent-driven or family-driven or caregiver-driven or community-driven).tw,kf. 3305

**4 1 or 2 or 3 561339 -- Concept 1: Patient Participation**

5 Infant/ or Child/ or Adolescent/ or Minors/ or Puberty/ or Pediatrics/ or Schools/ or Child health/ or Adolescent health/ 3632371

6 (newborn* or "new born?" or new-born or neonat* or neo-nat* or preterm or prematur* or preemie* or NICU or NICUs or PICU or PICUs or infan* or baby or babies or toddler* or child* or adolescen* or teen* or preteen* or pre-teen* or youth* or pediatric* or paediatric* or "young people" or "young person" or minor or juvenile* or boy* or girl* or kid or kids).tw,kf. 3411743

**7 5 or 6 5200071 – Concept 2: Pediatric Population**

8 Biomedical research/ or Research Design/ 205304

9 ((biomedical or experimental or investigate* or medical or health) adj3 (research or study or studies)).tw,kf. 1377220

**10 8 or 9 1555523 – Concept 3: Biomedical Research**

**11 4 and 7 and 10 18753 – Concept 1 + 2 + 3 Combined**

12 meta-analysis/ or "systematic review"/ or network meta-analysis/ or meta-analysis as topic/ or "Review Literature as Topic"/ 383899

13 ((systematic* adj3 (review* or overview*)) or (methodologic* adj2 (review* or overview*))).ti,ab,kf,kw. 369007

14 ((scoping or narrative or rapid) adj3 (review* or overview*)).ti,ab,kw,kf. 71905

15 (systematic review or meta-analysis).pt. 353271

16 ((integrative adj3 (review* or overview*)) or (collaborative adj3 (review* or overview*)) or (pool* adj3 analy*)).ti,ab,kf,kw. 43035

17 (handsearch* or hand search*).ti,ab,kf,kw. 11655

18 (meta analy* or metanaly* or meta regression* or metaregression*).ti,ab,kf,kw. 313299

19 (medline or cochrane or pubmed or medlars or embase or cinahl or PsycINFO).ti,ab,hw. 415730

20 umbrella review*.ti,ab,kf. 2099

21 (cochrane or systematic review?).jw. 21060

**22 or/12-21 748789 – Adapted Reviews Hedge from CADTH***

23 11 and 22 1715

**24 limit 23 to (english language and yr="2009 -Current") 1565**

**25 ("25323964" or "31700676" or "34106978" or "27516003" or "36179891").ui. 5**

**Seed Articles**

**26 24 or 25 1569 – All 5 seed articles captured.**

* Adapted from CADTH: _SR / MA / HTA / ITC - MEDLINE, Embase, PsycInfo. In: CADTH Search Filters Database. Ottawa: CADTH; 2024: <https://searchfilters.cadth.ca/link/33>. Accessed 2024-07-09.
