# Supplementary material for: Data Sources and Analytic Approaches Used to Evaluate the Impact of Patient and Public Involvement in Child Health Research: Simple Random Survey of Individual Studies in Published Reviews
Source: Health Expect. 2026 Feb 20;29(1):e70577. doi: 10.1111/hex.70577 (PMC12928104; doi:10.1111/hex.70577)
Supplement: Supplementary file 2 — Supplementary Table 2: Data Abstraction Form. [file HEX-29-e70577-s001.docx]

# Supplementary Table 2. Data Abstraction Form

# DATA ABSTRACTION FORM

| SECTION 1. INCLUSION CRITERIA | | | |
| --- | --- | --- | --- |
|  | | Yes | No |
| Is the paper a published, peer-reviewed, empirical study? | |  |  |
| Is it a single study (i.e. not a review)? | |  |  |
| Does the study population include participants under the age of 18? | |  |  |
| Is there patient & family engagement reported in the primary study? | |  |  |
| Is the patient and family engagement in research? | |  |  |
| Is at least one impact of engagement reported? | |  |  |
| SECTION 2. “PRIMARY STUDY” CHARACTERISTICS | | | |
| Year of Publication: | | | |
| Journal Impact Factor (web of science): | | | |
| Country of Origin (where the study was conducted): | | | |
| Multi-site study? | Yes No | | |
| Multi-country study? | Yes No | | |
| Study Design: | RCT Quasi-experimental  Cohort  Case-control  Delphi study  Cross-sectional survey  Case series  Mixed-methods  Qualitative | | |
| Study Setting: | Hospital – in or outpatient MD Primary Care/Alternative therapy  Community  Population-level | | |
| Clinical Topic | | Allergy & intolerance | | --- | | Blood Disorders | | Cancer | | Child Health | | Complementary & alternative medicine | | Consumer & communication strategies | | Dentistry & oral health | | Developmental, psychosocial & learning problems | | Diagnosis | | Ear, nose & throat | | Effective practice & health systems | | Endocrine & metabolic | | Eyes & vision | | Gastroenterology & hepatology | | Genetic disorders | | Gynaecology | | Health & safety at work | | Health professional education  Heart and circulation | | Infectious disease | | Insurance medicine | | Kidney disease | | Lung & airways | | Mental health | | Methodology | | Neonatal care | | Neurology | | Orthopaedics & trauma | | Pain & anaesthesia | | Pregnancy & childbirth | | Public health | | Reproductive & sexual health | | Rheumatology | | Skin disorders | | Tobacco, drugs & alcohol | | Urology | | Wounds  Other: | | | |
| Age range of study participants Multi-select | Child (0-11) Youth (12-17)  Parents | | |
| Median age of study participants (if provided) |  | | |
| Sample size (total) |  | | |
| Primary outcome specified? | Specified Not specified 🡪 proceed to section 3 | | |
| Primary outcome type | Clinical Biomedical | | |
| Primary outcome PROM? | Yes No | | |
| Multiple primary outcomes? | Yes No | | |
| Primary outcome statistically significant difference between groups? | Yes No | | |
|  | | | |
| SECTION 3. PATIENT ENGAGEMENT CHARACTERISTICS | | | |
| Individuals engaged as partners Multi-select | Children (0-11 years)Youth (12-18 years) Representatives | | |
| Types of representatives? Multi-select | Parents/caregivers/guardians Other family members (e.g. siblings)  Professionals, eg, advocacy group member  Community members | | |
| Number of patient/family/community representatives engaged in research? Total | 1  2 – 5  6 – 10  11 – 20  21 – 50  51 – 99  100+ | | |
| Methods of engagement Multi-select | Patient-led  Co-PI  Advisory group Focus group Interview  Surveys/Written feedback | | |
| Stage of engagement Multi-select | Prior to or at the start of research During research  After the research was complete | | |
| Area of engagement | | 1. Priority setting 2. Development of research proposal 3. Funding application 4. Ethics application 5. Development of the research question | | --- | | 1. Selection of outcomes (including core outcomes set development) | | 1. Intervention development and refinement | | 1. Input on study design 2. Input on study conduct 3. Input on study materials | | 1. Input on approach to recruitment of participants | | 1. Intervention delivery | | 1. Data collection | | 1. Data analysis/results interpretation 2. Drafting manuscript | | 1. Dissemination and implementation of results | | | |
| Level of engagement (IAP2) | Consult – feedback gathered  Involve – input gathered throughout process but without any say in final decision making  Collaborate – patients/families engaged are partners, have some decision-making power  Empower – patients/families engaged have final say in decision making | | |
|  | | | |
| SECTION 4. EVALUATION OF IMPACT OF PATIENT ENGAGEMENT | | | |
| Does the study use a patient engagement reporting tool (e.g., GRIPP2)? | No Yes | | |
| If yes, which tool? |  | | |
| Impact evaluationdata sources **direct = group/interview conducted for the purpose of evaluating the impact of PPI*  **indirect = group/interview conducted to inform the primary study, used secondarily as data to evaluate impact* | Survey  Transcript of Face-to-Face (focus groups, interviews) – direct  Transcript of Face-to-Face (focus groups, interviews) – indirect  Field notes/researcher observation | | |
| Impact evaluation analytic approaches | Patient Partner anecdote (i.e. patient partner perception)  Researcher anecdote – research team (i.e. researcher perception)  Researcher anecdote – independent researcher/expert  Qualitative (content/thematic) analysis  Quantitative analysis  Mixed methods | | |
| Impact on research process †Multiple selections possible. | 1. Helping to identify/prioritize important/relevant topics for research 2. Refining the research question 3. Co-producing study materials appropriate for patient population 4. Improving design of research 5. Improving research efficiency 6. Helping to secure ethical approval 7. Helping to secure funding 8. Increasing participant recruitment/retention 9. Improving the usefulness/relevance of findings 10. Improving research quality 11. Enhanced analysis 12. Enhanced dissemination and implementation of research | | |
| Impact on research team †Multiple selections possible. | 1. Improved relationships between researchers and patients/families 2. Enhanced knowledge/skills 3. Improved provision of care 4. Networking/community building 5. Cultural competency/change in attitudes towards patient population 6. Increased need for resources 7. Gaining a broader perspective | | |
| Impact on patients and families †Multiple selections possible. | 1. Improved relationships between patients/families and researchers 2. Increase in knowledge and skills 3. Improved care experience or understanding of condition 4. Community building 5. Increased need for resources 6. Networking/career and academic outcomes 7. Realization of role that they can play to influence change/empowerment 8. Gaining a broader perspective 9. Improved relationship/understanding between parents and children 10. Increased resilience/independence | | |
