# Supplementary material for: Data Sources and Analytic Approaches Used to Evaluate the Impact of Patient and Public Involvement in Child Health Research: Simple Random Survey of Individual Studies in Published Reviews
Source: Health Expect. 2026 Feb 20;29(1):e70577. doi: 10.1111/hex.70577 (PMC12928104; doi:10.1111/hex.70577)
Supplement: Supplementary file 3 — Supplementary Table 3: Eligible Reviews. [file HEX-29-e70577-s003.docx]

**Supplementary Table 3.** Eligible Reviews

1. Anyon Y, Bender K, Kennedy H, Dechants J. A Systematic Review of Youth Participatory Action Research (YPAR) in the United States: Methodologies, Youth Outcomes, and Future Directions. Health Educ Behav. 2018 Dec;45(6):865–78.
2. Bailey S, Boddy K, Briscoe S, Morris C. Involving disabled children and young people as partners in research: a systematic review. Child. 2015 Jul;41(4):505–14.
3. Fløtten KJØ, Guerreiro AIF, Simonelli I, Solevåg AL, Aujoulat I. Adolescent and young adult patients as co‐researchers: A scoping review. Health Expectations. 2021 Aug;24(4):1044–55.
4. Flynn R, Walton S, Scott SD. Engaging children and families in pediatric Health Research: a scoping review. Res Involv Engagem. 2019 Dec;5(1):32.
5. Fountain S, Hale R, Spencer N, Morgan J, James L, Stewart MK. A 10-Year Systematic Review of Photovoice Projects With Youth in the United States. Health Promotion Practice. 2021 Nov;22(6):767–77.
6. Ibitoye BM, Garrett B, Ranger M, Stinson J. Conducting Patient-Oriented Research in Low-Income and Middle-Income Countries: A Scoping Review. Patient. 2023 Jan;16(1):19–29.
7. Kennedy H, DeChants J, Bender K, Anyon Y. More than Data Collectors: A Systematic Review of the Environmental Outcomes of Youth Inquiry Approaches in the United States. American J of Comm Psychol. 2019 Mar;63(1–2):208–26.
8. McCabe E, Amarbayan M (Megan), Rabi S, Mendoza J, Naqvi SF, Thapa Bajgain K, et al. Youth engagement in mental health research: A systematic review. Health Expectations. 2023 Feb;26(1):30–50.
9. Rouncefield-Swales A, Harris J, Carter B, Bray L, Bewley T, Martin R. Children and young people’s contributions to public involvement and engagement activities in health-related research: A scoping review. Evans CJ, editor. PLoS ONE. 2021 Jun 9;16(6):e0252774.
10. Shen S, Doyle‐Thomas KAR, Beesley L, Karmali A, Williams L, Tanel N, et al. How and why should we engage parents as co‐researchers in health research? A scoping review of current practices. Health Expectations. 2017 Aug;20(4):543–54.
11. Thomas C, Cockcroft E, Jenkins G, Liabo K. Working with children and young people in research: Supportive practices and pathways to impact. J Child Health Care. 2025 Mar;29(1):34–52.
12. Tschida JE, Lee JD, Pomales‐Ramos A, Koo V. Reported quality indicators and implementation outcomes of community partnership in autism intervention research: A systematic review. Autism Research. 2024 Feb;17(2):215–33.
13. Valdez ES, Skobic I, Valdez L, O Garcia D, Korchmaros J, Stevens S, et al. Youth Participatory Action Research for Youth Substance Use Prevention: A Systematic Review. Substance Use & Misuse. 2020 Jan 1;55(2):314–28.
14. Van Schelven F, Boeije H, Mariën V, Rademakers J. Patient and Public Involvement of young people with a chronic condition in projects in health and social care: A scoping review. Health Expectations. 2020 Aug;23(4):789–801.
15. Vanderhout SM, Bhalla M, Van A, Fergusson DA, Potter BK, Karoly A, et al. The Impact of Patient and Family Engagement in Child Health Research: A Scoping Review. The Journal of Pediatrics. 2023 Feb;253:115–28.
