# Supplementary material for: Data Sources and Analytic Approaches Used to Evaluate the Impact of Patient and Public Involvement in Child Health Research: Simple Random Survey of Individual Studies in Published Reviews
Source: Health Expect. 2026 Feb 20;29(1):e70577. doi: 10.1111/hex.70577 (PMC12928104; doi:10.1111/hex.70577)
Supplement: Supplementary file 4 — Supplementary Table 4: Citations for 100 primary studies randomly selected from 15 eligible reviews. [file HEX-29-e70577-s004.docx]

**Supplementary Table 4.** Citations for 100 primary studies randomly selected from 15 eligible reviews

1. Arches J. The role of groupwork in social action projects with youth. GPWK. 2012 Dec 26;22(1):59–77.
2. Balvanz P, Dodgen L, Quinn J, Holloway T, Hudspeth S, Eng E. From Voice to Choice: African American Youth Examine Childhood Obesity in Rural North Carolina. Prog Community Health Partnersh. 2016;10(2):293–303.
3. Berg M, Coman E, Schensul JJ. Youth Action Research for Prevention: A Multi‐level Intervention Designed to Increase Efficacy and Empowerment Among Urban Youth. American J of Comm Psychol. 2009 Jun;43(3–4):345–59.
4. Brady LM, Templeton L, Toner P, Watson J, Evans D, Percy-Smith B, et al. Involving young people in drug and alcohol research. DAT. 2018 Mar 5;18(1):28–38.
5. Cammarota J, Romero A. Participatory Action Research for High School Students: Transforming Policy, Practice, and the Personal With Social Justice Education. Educational Policy. 2011 May;25(3):488–506.
6. Cardarelli KM, Paul M, May B, Dunfee M, Browning S, Schoenberg N. “Youth Are More Aware and Intelligent than Imagined”: The Mountain Air Youth Photovoice Project. International Journal of Environmental Research and Public Health. 2019 Jan;16(20):3829.
7. Chappell P, Rule P, Dlamini M, Nkala N. Troubling power dynamics: Youth with disabilities as co-researchers in sexuality research in South Africa. Childhood. 2014 Aug 1;21(3):385–99.
8. Chopel A, Lee RE, Ortiz-Matute E, Peoples N, Homer Vagadori K, Curtis A, et al. The META-Oak Project: Using Photovoice to Investigate Youth Perspectives on Tobacco Companies’ Marketing of E-Cigarettes Toward Adolescents in Oakland. SAGE Open. 2019 Jul;9(3):215824401985742.
9. Christens BD, Dolan T. Interweaving Youth Development, Community Development, and Social Change Through Youth Organizing. Youth & Society. 2011 Jun;43(2):528–48.
10. Cleverley K, McCann E, O’Brien D, Davies J, Bennett K, Brennenstuhl S, et al. Prioritizing core components of successful transitions from child to adult mental health care: a national Delphi survey with youth, caregivers, and health professionals. Eur Child Adolesc Psychiatry. 2022 Nov;31(11):1739–52.
11. Conner JO, Strobel K. Leadership Development: An Examination of Individual and Programmatic Growth. Journal of Adolescent Research. 2007 May;22(3):275–97.
12. Coyne I, Prizeman G, Sheehan A, Malone H, While AE. An e-health intervention to support the transition of young people with long-term illnesses to adult healthcare services: Design and early use. Patient Educ Couns. 2016 Sep;99(9):1496–504.
13. d’Udekem Y, Forsdick V, Du Plessis K. Involvement of patients and parents in research undertaken by the Australian and New Zealand Fontan Registry. Cardiol Young. 2018 Apr;28(4):517–21.
14. Dennehy R, Cronin M, Arensman E. Involving young people in cyberbullying research: The implementation and evaluation of a rights‐based approach. Health Expectations. 2019 Feb;22(1):54–64.
15. Dixon-Woods M, Tarrant C, Jackson CJ, Jones DR, Kenyon S. Providing the results of research to participants: a mixed-method study of the benefits and challenges of a consultative approach. Clin Trials. 2011 Jun;8(3):330–41.
16. Edwards M, Lawson C, Rahman S, Conley K, Phillips H, Uings R. What does quality healthcare look like to adolescents and young adults? Ask the experts! Clin Med (Lond). 2016 Apr;16(2):146–51.
17. Edwards V, Wyatt K, Logan S, Britten N. Consulting parents about the design of a randomized controlled trial of osteopathy for children with cerebral palsy: Consulting parents about trial design. Health Expectations. 2011 Dec;14(4):429–38.
18. Elberse JE, Caron-Flinterman JF, Broerse JEW. Patient-expert partnerships in research: how to stimulate inclusion of patient perspectives. Health Expect. 2011 Sep;14(3):225–39.
19. Ennals P, Lessing K, Spies R, Egan R, Hemus P, Droppert K, et al. Co‐producing to understand what matters to young people living in youth residential rehabilitation services. Early Intervention Psych. 2022 Jul;16(7):782–91.
20. Flicker S, Skinner H, Read S, Veinot T, McClelland A, Saulnier P, et al. Falling Through the Cracks of the Big Cities. Can J Public Health. 2005 Jul;96(4):308–12.
21. Foster V, Young A. Reflecting on participatory methodologies: research with parents of babies requiring neonatal care. International Journal of Social Research Methodology. 2015 Jan 2;18(1):91–104.
22. Foster-Fishman PG, Law KM, Lichty LF, Aoun C. Youth ReACT for Social Change: A Method for Youth Participatory Action Research. American J of Comm Psychol. 2010 Sep;46(1–2):67–83.
23. Galletta A, Jones V. “Why are you doing this?” Questions on Purpose, Structure, and Outcomes in Participatory Action Research Engaging Youth and Teacher Candidates. Educational Studies. 2010 Jun 2;46(3):337–57.
24. Graham N, Mandy A, Clarke C, Morriss-Roberts C. Using children and young people as advocates to inform research design. British Journal of Occupational Therapy. 2017 Nov 1;80(11):684–8.
25. Griesemer I, Staley BS, Lightfoot AF, Bain L, Byrd D, Conway C, et al. Engaging Community Stakeholders in Research on Best Practices for Clinical Genomic Sequencing. Per Med. 2020 Nov;17(6):435–44.
26. Griffiths FE, Armoiry X, Atherton H, Bryce C, Buckle A, Cave JA, et al. The role of digital communication in patient–clinician communication for NHS providers of specialist clinical services for young people [the Long-term conditions Young people Networked Communication (LYNC) study]: a mixed-methods study [Internet]. Southampton (UK): NIHR Journals Library; 2018 [cited 2025 Feb 11]. (Health Services and Delivery Research). Available from: <http://www.ncbi.nlm.nih.gov/books/NBK482045/>
27. Groot B, Dedding C, Slob E, Maitland H, Teunissen T, Rutjes N, et al. Adolescents’ experiences with patient engagement in respiratory medicine. Pediatric Pulmonology. 2021 Jan;56(1):211–6.
28. Hunt A, Brown E, Coad J, Staniszewska S, Hacking S, Chesworth B, et al. “Why does it happen like this?” Consulting with users and providers prior to an evaluation of services for children with life limiting conditions and their families. J Child Health Care. 2015 Sep;19(3):320–33.
29. Irizarry J. Buscando la Libertad: Latino Youths in Search of Freedom in School. Democracy and Education [Internet]. 2011 Apr 29;19(1). Available from: <https://democracyeducationjournal.org/home/vol19/iss1/4>
30. Jardine CG, James A. Youth researching youth: benefits, limitations and ethical considerations within a participatory research process. Int J Circumpolar Health. 2012 May 8;71(0):1–9.
31. Jesús AD, Oviedo S, Feliz S. Global Kids Organizing in the Global City: Generation of Social Capital in a Youth Organizing Program.
32. Jurkowski JM, Green Mills LL, Lawson HA, Bovenzi MC, Quartimon R, Davison KK. Engaging Low-Income Parents in Childhood Obesity Prevention from Start to Finish: A Case Study. J Community Health. 2013 Feb;38(1):1–11.
33. Kendal SE, Milnes L, Welsby H, Pryjmachuk S, Co‐Researchers’ Group. Prioritizing young people’s emotional health support needs via participatory research. Psychiatric Ment Health Nurs. 2017 Jun;24(5):263–71.
34. Kirshner B, Pozzoboni K, Jones H. Learning how to manage bias: A case study of youth participatory action research. Applied Developmental Science. 2011;15(3):140–55.
35. Kirshner B, Pozzoboni KM. Student Interpretations of a School Closure: Implications for Student Voice in Equity-Based School Reform. Teachers College Record: The Voice of Scholarship in Education. 2011 Aug;113(8):1633–67.
36. Kramer JM, Schwartz AE. Development of the Pediatric Disability Inventory-Patient Reported Outcome (PEDI-PRO) measurement conceptual framework and item candidates. Scand J Occup Ther. 2018 Sep;25(5):335–46.
37. Kroeger S, Burton C, Comarata A, Combs C, Hamm C, Hopkins R, et al. Student Voice and Critical Reflection: Helping Students at Risk. TEACHING Exceptional Children. 2004 Jan;36(3):50–7.
38. Kulbok PA, Meszaros PS, Bond DC, Thatcher E, Park E, Kimbrell M, et al. Youths As Partners in a Community Participatory Project for Substance Use Prevention. Family & Community Health. 2015 Jan;38(1):3–11.
39. Lightfoot AF, Thatcher K, Simán FM, Eng E, Merino Y, Thomas T, et al. “What I wish my doctor knew about my life”: Using photovoice with immigrant Latino adolescents to explore barriers to healthcare. Qual Soc Work. 2019 Jan;18(1):60–80.
40. Lundy L, McEvoy L, Byrne B. Working With Young Children as Co-Researchers: An Approach Informed by the United Nations Convention on the Rights of the Child. Early Education & Development. 2011 Sep;22(5):714–36.
41. Maglajlić RA, Tiffany J. Participatory Action Research with Youth in Bosnia and Herzegovina. In: Youth Participation and Community Change. Routledge; 2006.
42. Malcolm C, Forbat L, Knighting K, Kearney N. Exploring the experiences and perspectives of families using a children’s hospice and professionals providing hospice care to identify future research priorities for children’s hospice care. Palliat Med. 2008 Dec;22(8):921–8.
43. Manning JC, Hemingway P, Redsell SA. Survived so what? Identifying priorities for research with children and families post-paediatric intensive care unit. Nurs Crit Care. 2018 Mar;23(2):68–74.
44. Marshall Z, Nixon S, Nepveux D, Vo T, Wilson C, Flicker S, et al. Navigating risks and professional roles: Research with lesbian, gay, bisexual, trans, and queer young people with intellectual disabilities. Journal of Empirical Research on Human Research Ethics. 2012;7(4):20–33.
45. McAnuff J, Brooks R, Duff C, Quinn M, Marshall J, Kolehmainen N. Improving participation outcomes and interventions in neurodisability: co-designing future research. Child Care Health Dev. 2017 Mar;43(2):298–306.
46. McIntyre A, Chatzopoulos N, Politi A, Roz J. Participatory action research: Collective reflections on gender, culture, and language. Teaching and Teacher Education. 2007 Jul 1;23(5):748–56.
47. Mellins CA, Nestadt D, Bhana A, Petersen I, Abrams EJ, Alicea S, et al. Adapting Evidence-Based Interventions to Meet the Needs of Adolescents Growing Up with HIV in South Africa: The VUKA Case Example. Glob Soc Welf. 2014 Sep 1;1(3):97–110.
48. Mitchell SJ, Slowther AM, Coad J, Akhtar S, Hyde E, Khan D, et al. Ethics and patient and public involvement with children and young people. Arch Dis Child Educ Pract Ed. 2019 Aug;104(4):195–200.
49. Mitchell S, Slowther AM, Coad J, Dale J. The journey through care: study protocol for a longitudinal qualitative interview study to investigate the healthcare experiences and preferences of children and young people with life-limiting and life-threatening conditions and their families in the West Midlands, UK. BMJ Open. 2018 Jan 21;8(1):e018266.
50. Mongeau S, Champagne M, Liben S. Participatory Research in Pediatric Palliative Care: Benefits and Challenges. J Palliat Care. 2007 Mar;23(1):5–13.
51. Morton KL, Atkin AJ, Corder K, Suhrcke M, Turner D, van Sluijs EMF. Engaging stakeholders and target groups in prioritising a public health intervention: the Creating Active School Environments (CASE) online Delphi study. BMJ Open. 2017 Jan 13;7(1):e013340.
52. Noonan J. When soda is a social justice issue: design and documentation of a participatory action research project with youth. Educational Action Research. 2015 Apr 3;23(2):194–206.
53. Noone J, Allen TL, Sullivan M, McKenzie G, Esqueda T, Ibarra N. Escuchando a Nuestros Jóvenes: a latino youth photovoice project on teen pregnancy. Hisp Health Care Int. 2014;12(2):63–70.
54. Noyes JP, Williams A, Allen D, Brocklehurst P, Carter C, Gregory JW, et al. Evidence into practice: evaluating a child-centred intervention for diabetes medicine management The EPIC Project. BMC Pediatr. 2010 Sep 27;10:70.
55. Oliver K, Rees R, Brady LM, Kavanagh J, Oliver S, Thomas J. Broadening public participation in systematic reviews: a case example involving young people in two configurative reviews. Res Synth Methods. 2015 Jun;6(2):206–17.
56. Ozer EJ, Newlan S, Douglas L, Hubbard E. “Bounded” Empowerment: Analyzing Tensions in the Practice of Youth‐Led Participatory Research in Urban Public Schools. American J of Comm Psychol. 2013 Sep;52(1–2):13–26.
57. Ozer EJ, Ritterman ML, Wanis MG. Participatory Action Research (PAR) in Middle School: Opportunities, Constraints, and Key Processes. American J of Comm Psychol. 2010 Sep;46(1–2):152–66.
58. Pavarini G, Lorimer J, Manzini A, Goundrey‐Smith E, Singh I. Co‐producing research with youth: The NeurOx young people’s advisory group model. Health Expectations. 2019 Aug;22(4):743–51.
59. Petteway RJ, Sheikhattari P, Wagner F. Toward an Intergenerational Model for Tobacco-Focused CBPR: Integrating Youth Perspectives via Photovoice. Health Promot Pract. 2019 Jan;20(1):67–77.
60. Phillips EN, Berg MJ, Rodriguez C, Morgan D. A Case Study of Participatory Action Research in a Public New England Middle School: Empowerment, Constraints and Challenges. American J of Comm Psychol. 2010 Sep;46(1–2):179–94.
61. Pinsker EA, Call KT, Tanaka A, Kahin AA, Dar SI, Ganey A, et al. The Development of Culturally Appropriate Tobacco Prevention Videos Targeted Toward Somali Youth. Prog Community Health Partnersh. 2017;11(2):129–36.
62. Poland BD, Tupker E, Breland K. Involving street youth in peer harm reduction education. The challenges of evaluation. Can J Public Health. 2002;93(5):344–8.
63. Powers LE, Garner T, Valnes B, Squire P, Turner A, Couture T, et al. Building a Successful Adult Life: Findings From Youth-Directed Research. Exceptionality. 2007 Mar 1;15(1):45–56.
64. Rahi JS, Tadić V, Keeley S, Lewando-Hundt G, Vision-related Quality of Life Group. Capturing children and young people’s perspectives to identify the content for a novel vision-related quality of life instrument. Ophthalmology. 2011 May;118(5):819–24.
65. Reich SM, Kay JS, Lin GC. Nourishing a Partnership to Improve Middle School Lunch Options: A Community-Based Participatory Research Project. Family & Community Health. 2015 Jan;38(1):77–86.
66. Rich C, Goncalves A, Guardiani M, O’Donnell E, Strzelecki J. Teen Advisory Committee: lessons learned by adolescents, facilitators, and hospital staff. Pediatr Nurs. 2014 Nov 1;40(6):289–96.
67. Rogers J, Morrell E, Enyedy N. Studying the Struggle: Contexts for Learning and Identity Development for Urban Youth. American Behavioral Scientist. 2007 Nov;51(3):419–43.
68. Rosen-Reynoso M, Kusminsky M, Gragoudas S, Putney H, Crossman MK, Sinclair J, et al. Youth-based participatory research: lessons learned from a transition research study. Pediatrics. 2010 Dec;126 Suppl 3:S177-182.
69. Ross L. Sustaining Youth Participation in a Long-term Tobacco Control Initiative: Consideration of a Social Justice Perspective. Youth & Society. 2011 Jun;43(2):681–704.
70. Samir N, Diaz AM, Hodgins M, Matic S, Bawden S, Khoury J, et al. Speaking Softly and Listening Hard: The Process of Involving Young Voices from a Culturally and Linguistically Diverse School in Child Health Research. Int J Environ Res Public Health. 2021 May 28;18(11):5808.
71. Sánchez P. “In Between Oprah and Cristina”: Urban Latina Youth Producing a Countertext with Participatory Action Research. Social Justice. 2009;36(4 (118)):54–68.
72. Saunders T, Mackie TI, Shah S, Gooding H, de Ferranti SD, Leslie LK. Young adult and parent stakeholder perspectives on participation in patient-centered comparative effectiveness research. J Comp Eff Res. 2016 Aug;5(5):487–97.
73. Schuch JC, de Hernandez BU, Williams L, Smith HA, Sorensen J, Furuseth OJ, et al. Por Nuestros Ojos: understanding social determinants of health through the eyes of youth. Prog Community Health Partnersh. 2014;8(2):197–205.
74. Scott MA, Pyne KB, Means DR. Approaching Praxis: YPAR as Critical Pedagogical Process in a College Access Program. hsj. 2015 Dec;98(2):138–57.
75. Sheikhan NY, Hawke LD, Cleverley K, Darnay K, Courey L, Szatmari P, et al. ‘It reshaped how I will do research’: A qualitative exploration of team members’ experiences with youth and family engagement in a randomized controlled trial. Health Expectations. 2021 Apr;24(2):589–600.
76. Shelef DQ, Rand C, Streisand R, Horn IB, Yadav K, Stewart L, et al. Using stakeholder engagement to develop a patient-centered pediatric asthma intervention. Journal of Allergy and Clinical Immunology. 2016 Dec;138(6):1512–7.
77. Smith L, Bratini L, Appio LM. “Everybody’s Teaching and Everybody’s Learning”: Photovoice and Youth Counseling. Jour of Counseling &amp; Develop. 2012 Jan;90(1):3–12.
78. Smith L, Davis K, Bhowmik M. Youth Participatory Action Research Groups as School Counseling Interventions. Professional School Counseling. 2010;14(2):174–82.
79. Snodin J, Bray L, Carter B, Jack B. Consulting with children, parents and a teacher to shape a qualitative study. Nurse Res. 2017 Sep 19;25(2):39–43.
80. Stahmer AC, Brookman-Frazee L, Rieth SR, Stoner JT, Feder JD, Searcy K, et al. Parent perceptions of an adapted evidence-based practice for toddlers with autism in a community setting. Autism. 2017 Feb;21(2):217–30.
81. Stanley LR, Kelly KJ, Swaim RC, Jackman D. Cultural Adaptation of the Be under Your Own Influence Media Campaign for Middle-School American Indian Youth. J Health Commun. 2018;23(12):1017–25.
82. Stuttaford M, Coe C. The “Learning” Component of Participatory Learning and Action in Health Research: Reflections From a Local Sure Start Evaluation. Qual Health Res. 2007 Dec 1;17(10):1351–60.
83. Suleiman AB, Soleimanpour S, London J. Youth Action for Health Through Youth-Led Research. Journal of Community Practice. 2006 Jan;14(1–2):125–45.
84. Taines C. Educational or Social Reform? Students Inform the Debate Over Improving Urban Schools. Education and Urban Society. 2012 May;44(3):247–73.
85. Tanjasiri SP, Lew R, Kuratani DG, Wong M, Fu L. Using Photovoice to Assess and Promote Environmental Approaches to Tobacco Control in AAPI Communities. Health Promot Pract. 2011 Sep;12(5):654–65.
86. Tanjasiri SP, Lew R, Mouttapa M, Lipton R, Lew L, Has S, et al. Environmental influences on tobacco use among Asian American and Pacific Islander youth. Health Promot Pract. 2013 Sep;14(5 Suppl):40S-7S.
87. Tsang VWL, Fletcher S, Thompson C, Smith S. A novel way to engage youth in research: evaluation of a participatory health research project by the international children’s advisory network youth council. International Journal of Adolescence and Youth. 2020 Dec 31;25(1):676–86.
88. Tume LN, Preston J, Blackwood B. Parents’ and young people’s involvement in designing a trial of ventilator weaning. Nursing in Critical Care [Internet]. 2016 May [cited 2025 Feb 11];21(3). Available from: <https://onlinelibrary.wiley.com/doi/10.1111/nicc.12221>
89. Turner KCN, Hayes NV, Way K. Critical Multimodal Hip Hop Production: A Social Justice Approach to African American Language and Literacy Practices. Equity & Excellence in Education. 2013 Jul;46(3):342–54.
90. Uding N, Kieckhefer GM, Trahms CM. Parent and community participation in program design. Clin Nurs Res. 2009 Feb;18(1):68–79.
91. Van Schelven F, Boeije H, Inhulsen MB, Sattoe J, Rademakers J. “We Know What We Are Talking about”: Experiences of Young People with a Chronic Condition Involved in a Participatory Youth Panel and Their Perceived Impact. Child Care in Practice. 2021 Apr 3;27(2):191–207.
92. Van Staa A, Jedeloo S, Latour JM, Trappenburg MJ. Exciting but exhausting: experiences with participatory research with chronically ill adolescents. Health Expectations. 2010;13(1):95–107.
93. Viksveen P, Cardenas NE, Ibenfeldt M, Meldahl LG, Krijger L, Game JR, et al. Involvement of adolescent representatives and coresearchers in mental health research: Experiences from a research project. Health Expectations. 2022 Feb;25(1):322–32.
94. Walker KC, Saito RN. You<th Are Here: Promoting Youth Spaces through Community Mapping. Afterschool Matters [Internet]. 2011 [cited 2025 Feb 11]; Available from: <https://eric.ed.gov/?id=EJ980183>
95. Walton K, Ambrose T, Annis A, Ma DWl, Haines J. Putting family into family-based obesity prevention: enhancing participant engagement through a novel integrated knowledge translation strategy. BMC Med Res Methodol. 2018 Dec;18(1):126.
96. Wells N, Bronheim S, Zyzanski S, Hoover C. Psychometric Evaluation of a Consumer-Developed Family-Centered Care Assessment Tool. Matern Child Health J. 2015 Sep;19(9):1899–909.
97. White D, Shoffner A, Johnson K, Knowles N, Mills M. Advancing Positive Youth Development: Perspectives of Youth as Researchers and Evaluators. JOE [Internet]. 2012 Aug 1 [cited 2025 Feb 11];50(4). Available from: <https://tigerprints.clemson.edu/joe/vol50/iss4/5/>
98. Wintels SC, Smits DW, van Wesel F, Verheijden J, Ketelaar M, PERRIN PiP Study Group. How do adolescents with cerebral palsy participate? Learning from their personal experiences. Health Expect. 2018 Dec;21(6):1024–34.
99. Wright DE, Mahiri J. Literacy Learning Within Community Action Projects for Social Change. J Adolescent &amp; Adult Lit. 2012 Oct;56(2):123–31.
100. Zenkov K, Pellegrino A, Sell C, Ewaida M, Bell A, Fell M, et al. Picturing Kids and “Kids” as Researchers: Preservice Teachers and Effective Writing Instruction for Diverse Youth and English Language Learners. The New Educator. 2014 Oct 2;10(4):306–30.
